# Simultaneous Renal Oncocytoma and Lymphoma: Interest of Lymphadenectomy

**DOI:** 10.1155/2013/729108

**Published:** 2013-07-28

**Authors:** Angela Orcurto, Benoît Lhermitte, Alain Sermier, Dominik Berthold

**Affiliations:** ^1^Oncology Service, University Hospital of Lausanne, Switzerland; ^2^Pathology Service, University Hospital of Lausanne, Switzerland; ^3^Urology Service, University Hospital of Lausanne, Switzerland

## Abstract

Kidney lesions may be difficult to diagnose only by radiological exams, often requiring proof by tissue biopsy. Moreover, if enlarged regional lymph nodes are also present, the spectrum of differential diagnoses is even greater. The role of regional lymph node dissection in this setting is not clearly established. We show the case of a patient with a kidney mass associated with a conglomerate of para-aortic and iliac lymphadenopathies corresponding to an oncocytoma and a nodular lymphocyte predominant Hodgkin' lymphoma, respectively. Diagnosis of these two lesions was performed by morphology and immunohistochemistry. This case reflects how imaging can mislead to diagnosis and how histological confirmation helps decide treatment management.

## 1. Introduction

Renal oncocytomas are uncommon benign neoplasms of the urinary tract, accounting for 3% to 9% of all renal tumors [1]. Most cases are found incidentally by radiological exams, as they are indolent and mostly asymptomatic. Distinguishing an oncocytoma from a malignant tumor only by imaging can be challenging. Biopsy remains the mainstay of diagnosis. Having tissue proven diagnosis permits, depending on tumor size, to choose active surveillance over surgical interventions and to preserve kidney function. There is no sufficient evidence in the literature on whether lymph node dissection should be done whenever a suspected kidney lesion is found.

## 2. Case Report

**Figure 1:**
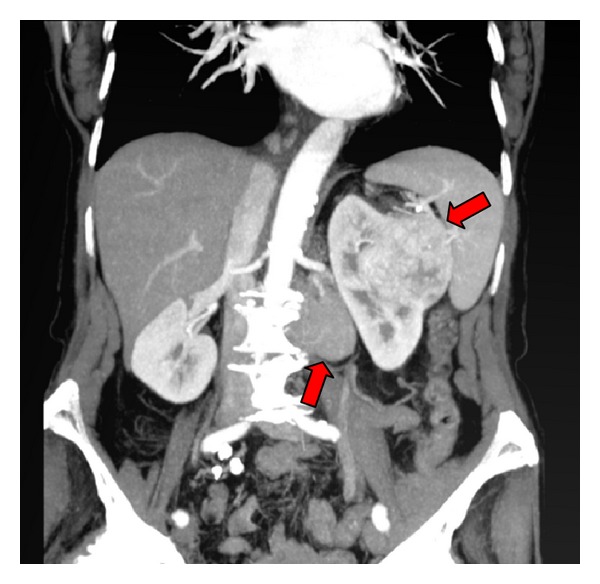
Left kidney lesion with left para-aortic lymphadenopathies.

A 76-year-old woman with no significant past medical history was admitted because of a new rapid atrial fibrillation and congestive heart failure due to an underlying coronary heart disease. A computed tomography (CT) was requested to rule out pulmonary embolism. Incidentally, a large left kidney mass was discovered. The CT showed the 9 cm diameter lesion localized in the midregion of the left kidney, with central areas of low attenuation and a conglomerate of left para-aortic and iliac lymphadenopathies (Figure 1). No other distant lesion was seen. 

The patient did not report any pain, weight loss, B symptoms, or haematuria. On physical examination, bibasal rales and bipedal pitting edema were noted. No red blood cells were found in the urinalysis. The hemoglobin, serum creatinine, and urea were also normal.

A malignant kidney tumor was suspected. The patient was addressed at our oncology department to discuss treatment. Because of the bulky lymphadenopathies, the urological surgeon did inquire about the role of neoadjuvant treatment. Considering the lack of histological diagnosis and the underlying heart disease, no indication for targeted treatment was retained, and a surgical resection was proposed. A total left nephrectomy with para-aortic lymphadenectomy by median laparotomy was performed. The anatomopathological report revealed a 8 × 8 × 7 cm tan-brown nonencapsulated tumor in the left kidney with central fibrosis. Histologically, well-differentiated neoplastic cells were seen with abundant eosinophilic granular cytoplasm, compatible with oncocytoma. The multiple para-aortic lymph nodes showed a nodular growth pattern and a background of lymphohistiocytes with a phenotype B CD20+. Complementary immunohistochemistry exams confirmed a nodular lymphocyte predominant Hodgkin' lymphoma (NLPHL). The staging evaluation concluded to a stage IIA for the NLPHL, and the oncocytoma showed no distant metastasis. Only clinical and radiologic followup was proposed.

## 3. Discussion

In our case, the initial radiological interpretation of the renal oncocytoma was challenging. Morphological characteristics were typical for renal cell carcinoma (RCC) as well, leading to prioritize a surgical resection for what was thought to be a malignant lesion. As retroperitoneal lymph nodes were voluminously present, a sampling lymphadenectomy was also performed. The anatomopathological report revealed two unexpected diagnoses.

There is no clear data in the literature concerning benefit of lymph node dissection in RCC. It is well known that the survival of patients with regional lymph node metastasis is similar to that of patients with distant metastatic lesions [2]. Thus, it is important to make a good staging of the disease in order to choose the best therapeutic approach. There is only one prospective randomized trial showing that lymph node dissection in clinical N0 cases has minimal benefit: Blom et al. randomized 772 patients between nephrectomy alone and nephrectomy with lymph node dissection. The rate of node positivity was low (4%), and no survival benefit was shown [3]. However, when lymph node metastasis is suspected, lymphadenectomy should be considered. Pantuck et al. reported in a retrospective study with 900 RCC patients treated surgically that, in node positive cases, lymphadenectomy was associated with improved survival and, moreover, with better response to immunotherapy.

This case illustrates how alone imaging can be misleading. A biopsy could have been performed, but that would not have avoided the nephrectomy, as the kidney lesion was voluminous. Lymph node resection permitted to diagnose a lymphoma instead of a metastatic state of the presumably RCC, changing completely the treatment approach. Lymph node dissection was indicated in this situation, because it revealed crucial diagnostic information. No oncologic treatment should be delivered based on assumptions without histological confirmation. Assessments in a multidisciplinary meeting including a medical oncologist, a urologist, and a radiologist can help to avoid regrettable pitfalls.
